# Quantitative Measurements of Stromal and Epithelial Riboflavin With a Novel Transepithelial High-Concentration Riboflavin Soak-and-Rinse Protocol

**DOI:** 10.1167/tvst.14.11.10

**Published:** 2025-11-12

**Authors:** Omkar C. Thaware, Elizabeth White, Reid Wilson, Shuibin Ni, Yifan Jian, Ted Acott, Yan Li, David Huang

**Affiliations:** 1 Casey Eye Institute, Oregon Health & Science University, Portland, OR, USA; 2 Department of Biomedical Engineering, Oregon Health & Science University, Portland, OR, USA; 3 Department of Chemical Physiology & Biochemistry, School of Medicine, Oregon Health & Science University, Portland, OR, USA

**Keywords:** keratoconus, corneal collagen crosslinking, transepithelial CXL, riboflavin

## Abstract

**Purpose:**

To enrich stromal riboflavin concentration and reduce epithelial riboflavin in transepithelial corneal collagen crosslinking (CXL).

**Methods:**

Ex vivo experiments on rabbit corneas were performed. The control group followed the standard (epi-off) Dresden CXL: a 30-minute epi-off application of 0.1% riboflavin and 20% dextran. The transepithelial riboflavin solutions consisted of various riboflavin concentrations in 1% hydroxypropyl methylcellulose in 0.45% saline, with or without 0.01% benzalkonium chloride (BAK). The novel soak-and-rinse protocol consists of 0.8% riboflavin with 0.01% BAK and 1% hydroxypropyl methylcellulose (hypotonic) applied for 20 minutes, followed by a 10-minute saline rinse. Stromal and epithelial thicknesses were measured by optical coherence tomography; riboflavin concentrations were quantified by spectrophotometry on 3-mm stromal buttons and epithelial eluates. Statistical analysis employed one-way analysis of variance, linear regression, and one-tailed unpaired *t*-tests.

**Results:**

The 20-minute soak increased stromal riboflavin compared to the 10-minute soak. A 76% higher stromal concentration was achieved by adding BAK to the transepithelial 0.8% riboflavin solution (*P* < 0.05). The 10-minute rinse achieved a Dresden-equivalent stromal riboflavin level and reduced epithelial riboflavin by 5.9-fold compared to a 20-second rinse (*P* < 0.0001).

**Conclusions:**

Stromal riboflavin concentrations equivalent to those achieved with the epi-off protocol can be achieved by transepithelial application of the novel high-concentration riboflavin formulation. The additional 10-minute rinse effectively reduced epithelial riboflavin levels, facilitating the delivery of ultraviolet light and oxygen into the stroma during CXL.

**Translational Relevance:**

The novel transepithelial riboflavin soak-and-rinse protocol may potentially enhance the efficacy of transepithelial CXL by reducing epithelial consumption of ultraviolet light and oxygen and increasing the stromal CXL reaction.

## Introduction

Keratoconus is characterized by progressive corneal thinning and curvature distortion that leads to decreased quality of vision. The prevalence of keratoconus is estimated to be 1.38 per 1000 people worldwide.^1^ Rigid contact lenses are commonly used to rehabilitate vision, and corneal transplantation may become necessary in severe cases. Corneal collagen crosslinking (CXL) was first introduced in 2003 to prevent keratoconus from progressing to severe stages.^2^ It halts keratoconus progression by creating additional covalent bonds within the corneal stroma. The photochemical reaction, which occurs during CXL, requires three essential agents: riboflavin, ultraviolet (UV) light, and O_2_.^2^^,^^3^ Riboflavin is a photosensitizer that facilitates the creation of reactive oxygen species (ROS) under 365-nm wavelength UV light. The ROS, in turn, creates covalent bonds between extracellular matrix molecules to strengthen the corneal stroma. The standard Dresden protocol includes central 9-mm epithelial removal (epi-off), followed by 30-minute 0.1% riboflavin soaking and 30-minute 3-mW/cm^2^ UV-A irradiation under normal air (21% O_2_).^2^ Although the standard CXL procedure has been to stabilize the corneal shape and visual acuity over the period of 7 to 10 years,^4^^,^^5^ the step of epithelial removal is associated with a significant risk of delayed epithelial healing. Studies have shown that delayed healing can lead to visually significant stromal haze^6^ and corneal infection, which often leads to scarring and visual loss. To reduce the risk of these complications, some practitioners perform CXL over the intact epithelium in clinical trials. However, an intact corneal epithelium acts as a barrier to the essential components of the CXL reaction. The epithelium reduces riboflavin diffusion into the stroma and creates an enriched layer of riboflavin anterior to the stroma that absorbs UV light and consumes O_2_, reducing their stromal penetration.^3^^,^^7^^,^^8^ For these reasons, currently, transepithelial CXL protocols are not as effective as epi-off CXL.^9^^,^^10^ There is a need for a transepithelial CXL protocol that is equivalent to or more effective than the epi-off Dresden protocol, which still has a significant rate (6%–8%) of treatment failure that requires either corneal transplantation or retreatment.^11^

**Figure 1.**
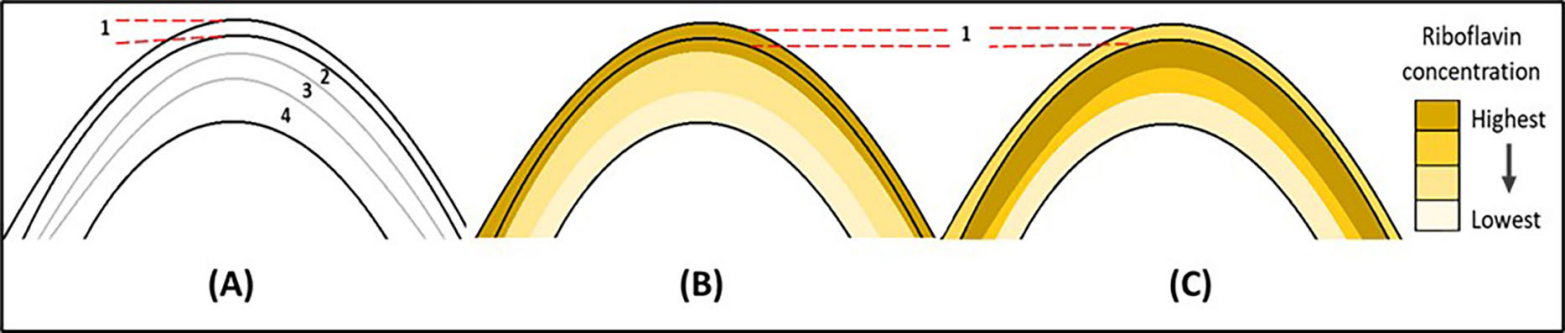
Graphical illustration of riboflavin concentration at different depths of the cornea in the soak-and-rinse protocol. (**A**) Corneal layers: 1, epithelium; 2, anterior stroma; 3, middle stroma; and 4, posterior stroma. (**B**) Corneal riboflavin distribution immediately after riboflavin soaking. (**C**) Corneal riboflavin distribution after soaking and then 10 minutes of rinsing.

The current study introduces a novel transepithelial high-concentration riboflavin formulation with a soak-and-rinse protocol. The protocol is hypothesized to boost riboflavin diffusion into the stroma through an intact epithelium while minimizing its epithelial concentration, as graphically represented in Figure 1. One aim of the new protocol is to use a high riboflavin concentration in the soaking solution to produce a high stromal riboflavin concentration similar to that of the standard epi-off riboflavin soaking. The second aim is to use rinsing to reduce the epithelial riboflavin concentration to reduce O_2_ consumption in the epithelium during UV irradiation, thereby enhancing stromal penetration of the CXL reactions.

## Materials and Methods

### Sample Preparation

Ex vivo experiments were performed with freshly enucleated New Zealand White rabbit eyes (average age 5 months old and weighing 2.5 kg) (Sierra for Medical Science, Whittier, CA, USA). Eyes were harvested and shipped overnight on ice in an insulated container (∼4°C) to perform all experimental procedures within 30 hours of harvesting. Corneas were divided into two major groups: active control and transepithelial (Table). According to the standard epi-off Dresden protocol, eyes in the control group received an application of an isotonic solution consisting of 0.1% riboflavin with 20% dextran. Riboflavin solutions were prepared using riboflavin 5-phosphate sodium (molecular weight = 478.33 g/mol). The osmolarity of isotonic solutions was measured as approximately 350 mOsmol/L. The transepithelial group received application of hypotonic solutions with various concentrations of riboflavin. All hypotonic solutions were prepared in 0.45% saline (NaCl) with 1% hydroxypropyl methylcellulose. Some solutions also contained 0.01% benzalkonium chloride (BAK) to enhance transepithelial riboflavin diffusion. The osmolarity of all hypotonic solutions was measured as approximately 155 mOsmol/L. The pH was titrated between 7.1 and 7.2 in all solutions.

**Table.** Riboflavin Protocols With Details of Epithelial Status, Riboflavin and Other Chemical Composition, and Soaking and Rinsing Period

Study Group	Riboflavin Formulation (Number of Corneas)	Soaking Period (One Drop Every 2 Minutes)	Rinsing Period
Control (epi-off)	0.1% riboflavin + 20% Dextran (6)	30 min	20 s (25–30 drops)
Transepithelial soak	0.25% riboflavin (4)	10 min and 20 min	
	0.5% riboflavin (2)		
	0.8% riboflavin (4)		
	0.8% riboflavin + 0.01% BAK (6)		
Transepithelial soak-and-rinse	0.8% riboflavin + 0.01% BAK (8)	20 min	20 s (25–30 drops) 10 min (initial 25–30 drops followed by 2 drops every 30–40 s)

### Experimental Procedures

The corneoscleral discs were carefully excised and mounted on an artificial anterior chamber filled with normal saline (0.9% NaCl) for the experimental procedures. Riboflavin soaking was performed by applying one drop of the riboflavin solution every 2 minutes in all protocols (Table).

In the control (epi-off soaking) group, the epithelium was removed from the central 9-mm-diameter area with a blunt hockey-stick spatula. The corneas were soaked in 0.1% riboflavin and 20% dextran solution for 30 minutes.

In the transepithelial soak and soak-and-rinse groups, the corneas were imaged with a 6 × 6-mm volumetric optical coherence tomography (OCT) scan to confirm intact epithelium at the beginning of the experiments.

In the transepithelial soak group, riboflavin soaking of two durations (10 vs. 20 minutes) and various riboflavin concentrations (0.25%, 0.5%, and 0.8%) were tested. The effect of 0.01% BAK was also tested with the 0.8% riboflavin solution.

The corneas in the soak-and-rinse protocol received 10 minutes of rinsing with two drops of normal saline every 30 seconds after 20 minutes of soaking with 0.8% riboflavin with BAK. This is in addition to the immediate rinse after soaking in groups with 20 to 30 drops of saline over 20 seconds.

Spectrophotometry (SpectraMax iD3; Molecular Devices, San Jose, CA, USA) was used to measure riboflavin concentration. Optical absorption at a 446-nm wavelength was measured. A calibration curve was constructed using measurements from gelatin samples with uniform thickness prepared with known riboflavin concentrations. The epithelium was removed from all corneas for the stromal riboflavin measurements. A central 7-mm cornea was immediately trephined with a Barron punch. The transstromal absorbance was measured on the central 3 mm of the stromal button. The stromal riboflavin concentration was calculated using the Beer–Lambert law; $A=\;ɛ*\bar{c}*l$, where *A* is absorbance measured at 446 nm, ε is the molar absorption coefficient, $\bar{c}$ is the overall stromal riboflavin concentration, and *l* is the average stromal button thickness of the central 3 mm measured under the OCT B-scan. The 24-well microplates were used for spectrophotometry measurements on both the gelatin sample curve and the stromal buttons. The absorbance of the empty microplate well and cornea stroma without riboflavin was subtracted from the measurements.

The epithelial riboflavin was measured after elution in a standardized volume of solution. Epithelial cells from the central 7 mm of the cornea were collected in 250 µL of phosphate buffer solution (PBS). The dilution factor was determined for individual samples based on the epithelial thickness of each cornea. The riboflavin-soaked cell-containing solution was left for 12 to 14 hours and stored in a dark container to avoid photobleaching before centrifugation at 750 × *g* for 5 minutes. Spectrophotometry was performed on a 105-µL supernatant sample.

### Statistical Analysis

One-way analysis of variance was used to assess the differences between topical riboflavin gradients. Bonferroni corrections were applied to *P* values to account for multiple comparisons. Linear regression models were used to determine the stromal riboflavin profile as the topical concentration increased for two soaking durations separately. Log values of both stromal and topical riboflavin concentrations were taken for normal distribution, and a Student's *t*-test was performed to determine differences in the slopes, as well as differences in the soaking period for individual protocols. A two-sample *t*-test was performed to determine the statistical significance between the two rinsing protocols for epithelial riboflavin concentration. All tests were performed using Excel (version 2402; Microsoft, Redmond, WA, USA) and the R statistical package (RStudio, version 4.2.2; R Project for Statistical Computing, Vienna, Austria).

## Results

**Figure 2.**
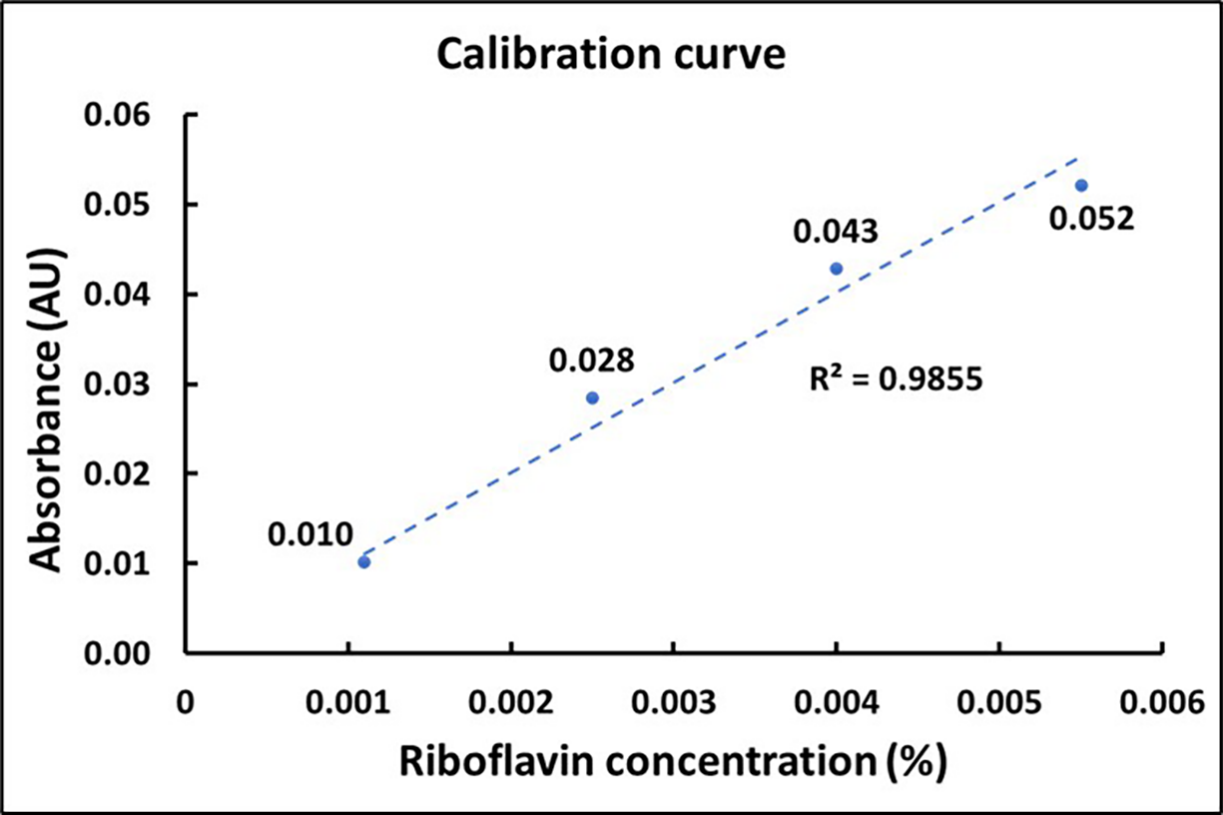
Different concentrations of riboflavin in gelatin samples were used to construct a calibration curve of optical absorbance at 446 nm. The linear fit was set to zero intercept. The absorbance of the gelatin sample without riboflavin was subtracted from the measurements.

**Figure 3.**
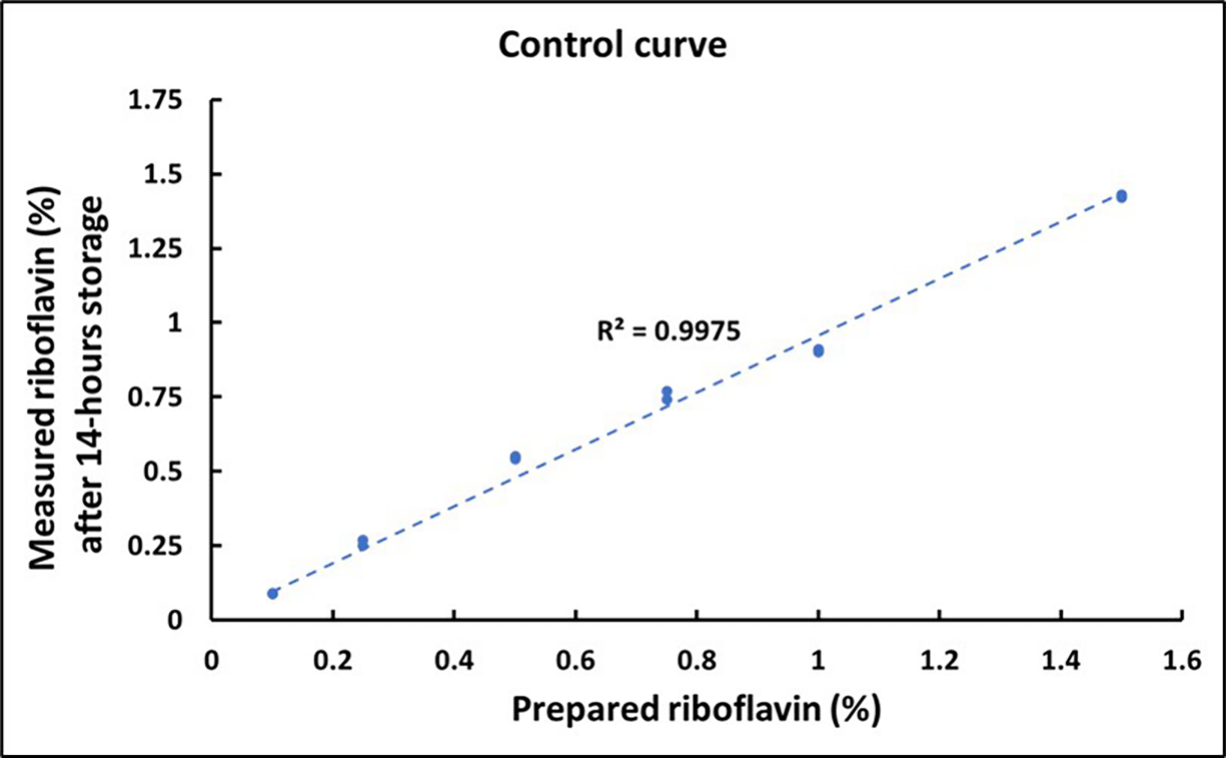
Control curve generated using known concentrations of riboflavin prepared in PBS, measured for optical absorbance at 446 nm. The absorbance of PBS without riboflavin was subtracted from all measurements. A linear fit was applied with the intercept constrained to zero.

The mean ± SD central epithelial thickness was 48 ± 5 µm, and the mean ± SD central stromal button thickness was measured as 464 ± 63 µm. Representative OCT B-scans illustrating epithelial and stromal button thickness measurements are shown in Supplementary Figure S1. The distributions of epithelial thickness and stromal button are presented in Supplementary Figure S2. All thickness measurements were obtained along the surface normal of the respective OCT B-scans. The mean ± SD baseline absorbance, averaged across three gelatin samples in a microplate well, was 0.034 ± 2.9 × 10^−5^. The gelatin samples were prepared with a uniform thickness of 335 µm. The baseline gelatin absorbance was deducted from riboflavin-treated gelatin samples for further analysis. The absorption coefficient of riboflavin (ε) was calculated as 12,422 M^−1^ cm^−1^ from the calibration curve shown in Figure 2, plotted using gelatin samples with known riboflavin concentration. The measured riboflavin absorption coefficient is very close to the value in the literature of 12,550 M^−1^ cm^−1^.^12^ The potential impact of photobleaching on fluorescence-based quantification was evaluated with a control experiment using a known concentration of riboflavin diluted in PBS, mimicking the dilution factor and measurement conditions of the stored epithelial cell–containing samples. The baseline absorbance through the PBS solution without riboflavin was deducted for further analysis. The control curve in Figure 3 shows minimal photobleaching at the tested riboflavin concentration, supporting the reliability of fluorescence measurements under the experimental protocol.

**Figure 4.**
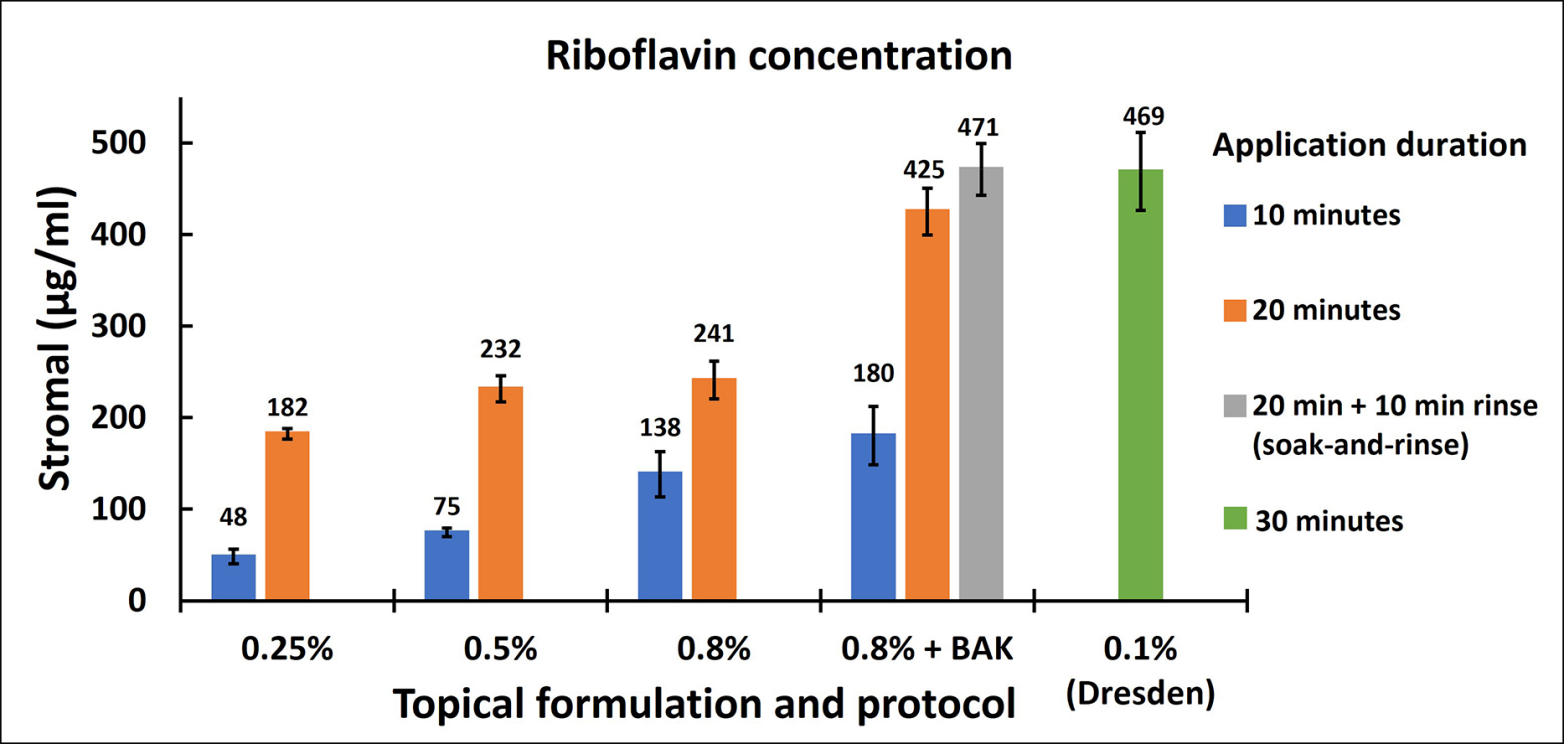
Stromal riboflavin concentration averaged over all corneas for respective riboflavin soaking protocols. Transepithelial soaking protocols with hypotonic (0.45%) saline and 1% hydroxypropyl methylcellulose were compared with epi-off soaking with the Dresden protocol (0.1% riboflavin in normal saline with 20% dextran for 30 minutes). Default rinsing is 20 seconds unless a 10-minute rinse is specified. The *error bar* represents the standard error of the respective riboflavin soaking protocols.

**Figure 5.**
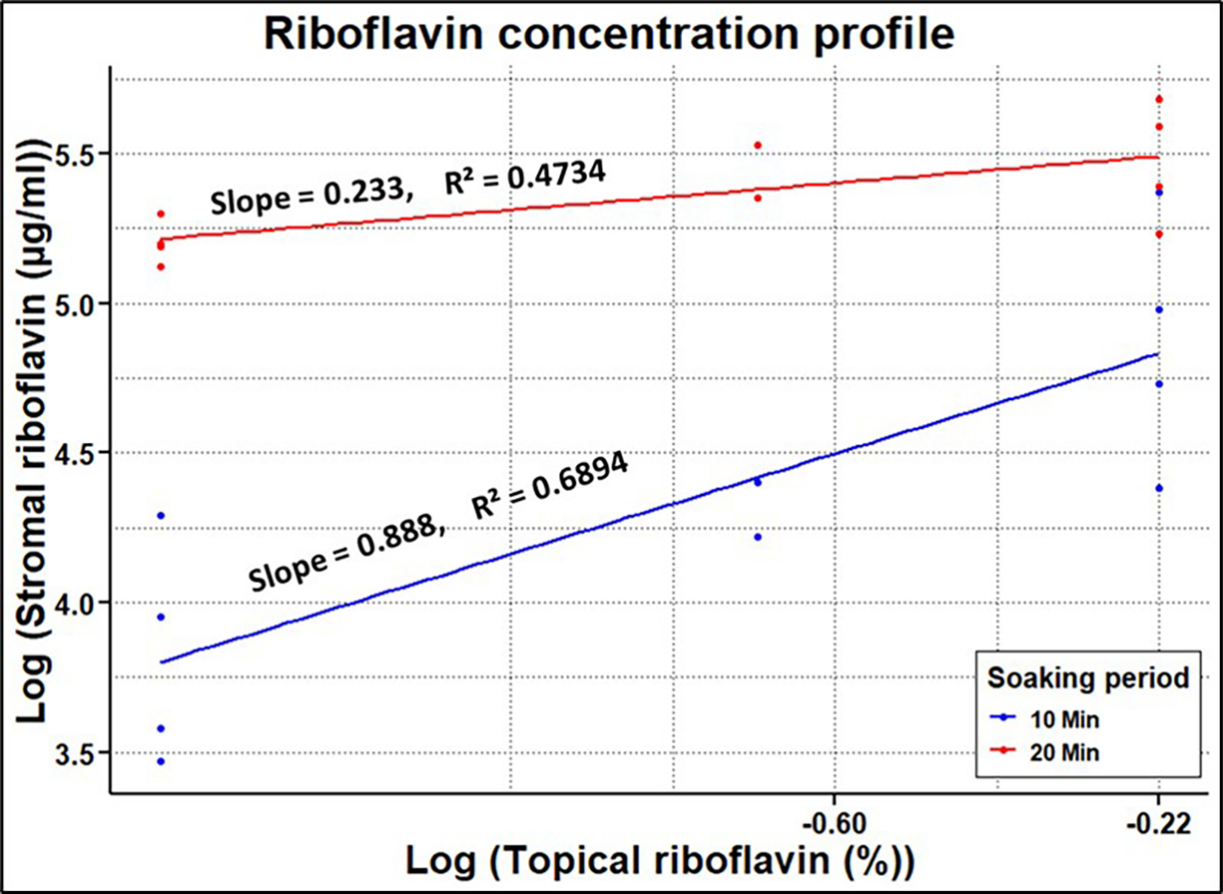
The stromal riboflavin concentration (µg/mL) plotted as a function of topical riboflavin concentration for 10-minute and 20-minute transepithelial soaks without BAK. The linear regression fits have the intercepts set to zero.

The stromal absorbance was normalized for a uniform stromal thickness of 1000 µm. The mean ± SD baseline stromal absorbance in the microplate well for two samples without riboflavin treatment was 1.18 ± 0.004. The baseline stromal absorbance was deducted from riboflavin-treated stroma for further analysis. The stromal riboflavin concentration from the experiments is shown in Figure 4. In the transepithelial soak group (no BAK), 20 minutes of soaking produced significantly (*P* < 0.05) greater stromal riboflavin concentration for all topical concentrations. Similarly, 0.8% topical riboflavin with BAK showed significantly (*P* < 0.05) greater stromal riboflavin with a 20-minute soak compared to a 10-minute soak. The linear regression model in Figure 5 shows the stromal riboflavin profile with respect to transepithelial topical riboflavin without BAK. The relationships between stromal riboflavin concentration and topical riboflavin concentration were not linear on a linear scale. However, the relationships were linear on a log scale (Fig. 4). For both 10- and 20-minute soaking, the stromal riboflavin increased with topical riboflavin concentration (*P* < 0.05).

**Figure 6.**
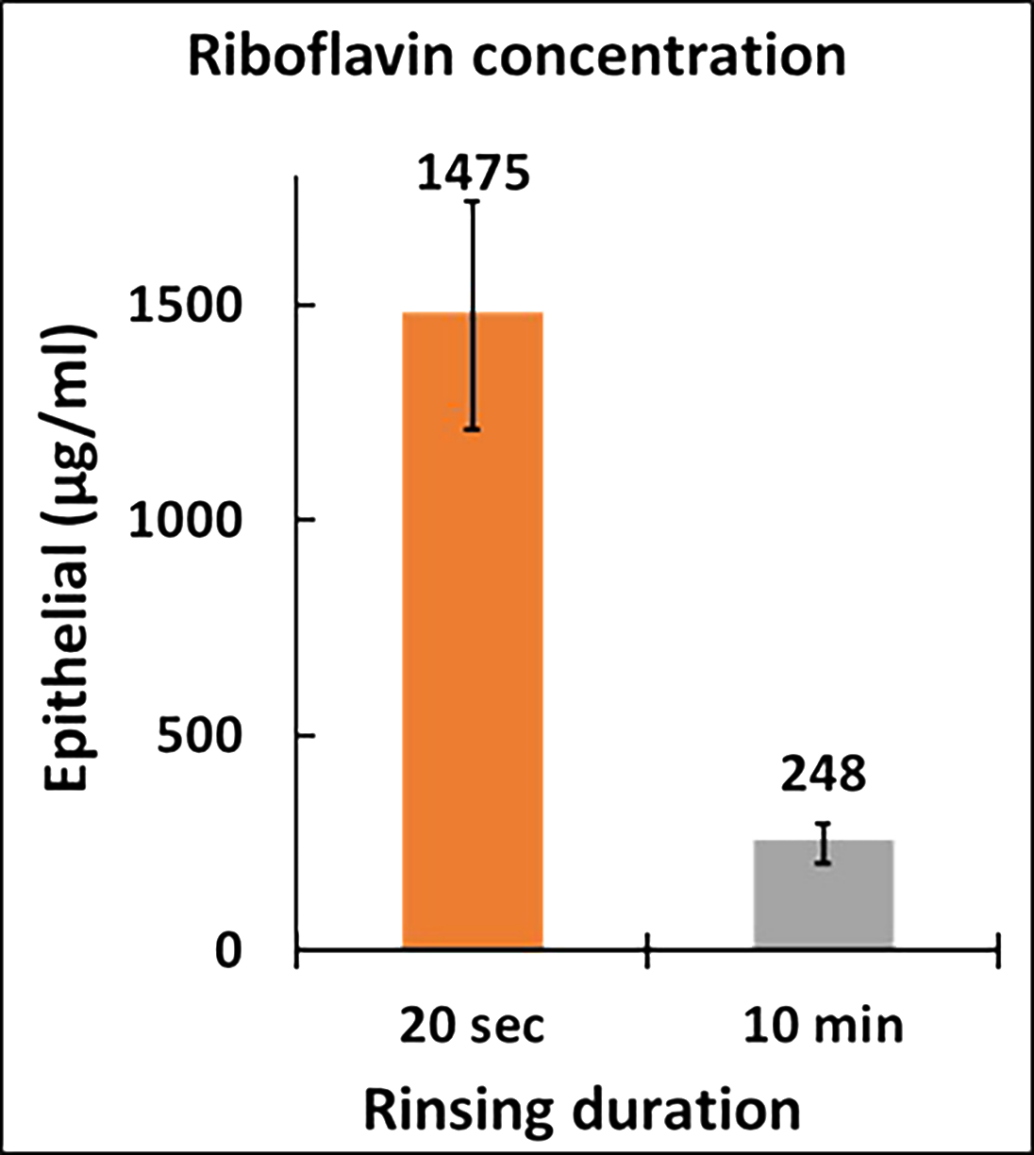
Epithelial riboflavin concentration averaged over all corneas for respective 20-second and 10-minute rinsing duration protocols. The transepithelial hypotonic solution of 0.8% riboflavin with 0.01% BAK was applied for the 20-minute soaking period. The *error bar* represents the standard error of the respective rinsing protocols.

By adding BAK to the 0.8% riboflavin solution, there was a 76% increase (*P* < 0.001) in the average stromal riboflavin for transepithelial soaking (Fig. 4). Ten minutes of rinsing further increased the stromal riboflavin concentration by 11% to a mean ± SE of 471 ± 29 µg/mL (Fig. 4). Although this increase was not statistically significant (*P* = 0.29), we can at least say that rinsing did not decrease the stromal riboflavin level. The 471-µg/mL result achieved by the 20-minute transepithelial 0.8% riboflavin + BAK soak-and-rinse was similar to the 469 ± 42 µg/mL stromal concentration produced by the standard Dresden epi-off protocol. Ten minutes of rinsing (Fig. 6) decreased the epithelial riboflavin concentration from a mean ± SE of 1475 ± 265 µg/mL to 248 ± 47 µg/mL. This 5.9-fold reduction was statistically significant (*P* < 0.001). With the 10-minute rinsing, the epithelial riboflavin concentration changed from 3.5 times the stromal concentration to 0.53 times the stromal concentration.

## Discussion

Multiple studies have shown the long-term efficacy of the standard epi-off CXL in stabilizing the progression of keratoconus.^4^^,^^5^ Hence, the epi-off CXL protocol can be considered an established standard for efficacy. It would be desirable for new transepithelial protocols to achieve a degree of stromal stiffening equivalent to or better than that achieved by current protocols. However, the literature has shown that the transepithelial approach has yet to show epi-off Dresden-equivalent biomechanical efficacy.^9^^,^^10^^,^^13^ This is likely due to the epithelial barrier to riboflavin and oxygen transport into the stroma.

The intact epithelium presents a significant barrier to riboflavin diffusion because of its lipophilic nature and intercellular tight junctions. An ex vivo study by Mastropasqua et al.^14^ has shown that four times greater stromal riboflavin was achieved with the standard epi-off riboflavin application compared to the transepithelial approach. The study by Mastropasqua et al.^14^ compared 0.1% riboflavin for an epi-off group with dextran (Ricrolin; Sooft Italia S.p.A., Montegiorgio, Italy) and the transepithelial group with dextran and ETDA (Ricrolin TE; Sooft Italia) for 20 and 30 minutes, respectively. Likewise, the current study showed about a 2.8-fold reduction in stromal riboflavin when 0.25% riboflavin was applied over intact epithelium compared to the epi-off protocol (Fig. 4).

Several approaches have been taken to enhance transepithelial riboflavin diffusion. One of the most common approaches is to increase the riboflavin concentration in the topical solution. Current clinical transepithelial protocols use topical concentrations up to 0.5%.^15^^–^^18^ A study by Rubinfeld et al.^16^ showed four times greater stromal riboflavin with a transepithelial solution of 0.5% riboflavin 5-phosphate (RiboStat; CXLO, Burlington, MA, USA) compared to a commercially available 0.25% transepithelial riboflavin solution. However, the two solutions in the study had different chemical compositions and application processes, making it impossible to assess the effect of topical riboflavin concentration on stromal concentration. On the other hand, by keeping the same chemical components and application methods, the current study showed a significant increase in stromal riboflavin as its concentration in the topical solution increased from 0.25% to 0.8% (Fig. 5). This significant increase was seen for both 10- and 20-minute application periods (*P* < 0.05). Hence, it provides a better understanding of the relationship between topical riboflavin and its respective stromal concentration.

In the one-dimensional diffusion model, the diffusion distance of riboflavin scales with topical application time.^19^ A study by Franke et al.^20^ found that the longer application time increases the absolute stromal riboflavin and the depth of the stromal riboflavin peak. We found that a 10-minute transepithelial soaking was insufficient to achieve the stromal concentration comparable to the Dresden protocol, but a 20-minute soaking sufficed. Most commercially available transepithelial CXL protocols suggest a soaking duration of between 10 and 20 minutes.^17^^,^^18^ Our study suggests 20 minutes are needed.

For riboflavin to be transported from the topical solution into the stroma, first, it must get past the epithelial barrier and then diffuse through the stromal depths. Chemical agents such as BAK and ETDA have been shown to enhance transepithelial riboflavin transport by loosening the tight junction between epithelial cells.^21^^–^^23^ The study by Kissner et al.^22^ on live rabbits found a significant increase in stromal riboflavin absorption when 0.02% BAK was added to a transepithelial riboflavin solution. Another study by Raiskup et al.^23^ showed a similar outcome with ex vivo rabbit eye experiments. The current study supports the findings of the experiments by Kissner et al.^22^ and Raiskup et al.,^23^ showing the 1.7-fold increase in the stromal riboflavin by adding 0.01% BAK to the 0.8% transepithelial riboflavin solution for a 20-minute application (*P* = 0.0001). The paracellular movement through the corneal epithelial barrier also depends on an osmotic gradient. Raiskup et al.^23^ also showed a twofold increase in riboflavin transport with a hypotonic (hypo-osmolar) solution compared to an isotonic solution. Hence, all transepithelial riboflavin solutions in the current study were prepared in a hypotonic solution with an osmolarity of 155 mOsmol/L. Iontophoresis is another approach to promote transepithelial riboflavin transport.^14^^,^^24^ However, it requires a cumbersome setup and has yet to achieve epi-off equivalent stromal riboflavin.^24^

The riboflavin in the epithelium is a barrier to stromal collagen crosslinking. It absorbs UV light and consumes O_2_ upon activation by UV, thereby reducing the available O_2_ that can diffuse into the stroma. This is critical as O_2_ transport limits the rate of free radical formation necessary for the CXL reaction. Thus, it is desirable to reduce the epithelial riboflavin concentration relative to the stroma. Our study results suggested that 10 minutes of saline rinsing was effective in achieving this goal—it reduced the epithelial riboflavin concentration by sixfold without decreasing the stromal concentration. The stromal concentration was not decreased presumably because for most of the 10 minutes of rinsing, the epithelial-stromal riboflavin gradient still drove riboflavin into the stroma rather than out.

Reduced riboflavin in the epithelium due to the soak-and-rise protocol should hypothetically minimize oxidative damage to epithelial cells during the photochemical reaction of CXL under UV exposure and oxygen supplementation. However, further in vivo studies are necessary to evaluate this hypothesis and more generally to assess the toxicity associated with various accelerated CXL protocols.

In conclusion, our results suggest that an optimal distribution of riboflavin can be achieved through a novel “soak-and-rinse” protocol and a topical formulation that combines high (0.8%) riboflavin concentration, hypotonicity, BAK, and a viscous carrier. This transepithelial protocol was able to achieve a stromal riboflavin concentration that was comparable to the standard epi-off Dresden protocol while reducing the epithelial riboflavin concentration to half of the stromal value. Although the present study was conducted ex vivo, the protocol is designed for clinical applicability and will be further evaluated in in vivo animal studies and, ultimately, in human clinical trials. We believe this novel protocol may facilitate effective transepithelial CXL, in combination with other advances such as oxygen supplementation.^25^^,^^26^ This hypothesis can be validated through additional investigations of stromal oxygen dynamics and biomechanical assessments using techniques such as optical coherence elastography. These investigations are underway to more fully assess the safety and translational potential of this protocol.

## Supplementary Material

Supplement 1

Supplement 2

## References

[1] Hashemi H, Heydarian S, Hooshmand E, et al. The prevalence and risk factors for keratoconus: a systematic review and meta-analysis. *Cornea*. 2020; 39(2): 263–270. 31498247 10.1097/ICO.0000000000002150

[2] Wollensak G, Spoerl E, Seiler T. Riboflavin/ultraviolet-A–induced collagen crosslinking for the treatment of keratoconus. *Am J Ophthalmol*. 2003; 135(5): 620–627. 12719068 10.1016/s0002-9394(02)02220-1

[3] Kamaev P, Friedman MD, Sherr E, Muller D. Photochemical kinetics of corneal cross-linking with riboflavin. *Invest Ophthalmol Vis Sci*. 2012; 53(4): 2360–2367. 22427580 10.1167/iovs.11-9385

[4] Kymionis GD, Grentzelos MA, Liakopoulos DA, et al. Long-term follow-up of corneal collagen cross-linking for keratoconus—the Cretan study. *Cornea*. 2014; 33(10): 1071–1079. 25170581 10.1097/ICO.0000000000000248

[5] Shaheen MS, Lolah MM, Piñero DP. The 7-year outcomes of epithelium-off corneal cross-linking in progressive keratoconus. *J Refract Surg*. 2018; 34(3): 181–186. 29522228 10.3928/1081597X-20180123-01

[6] Raiskup F, Hoyer A, Spoerl E. Permanent corneal haze after riboflavin-UVA-induced cross-linking in keratoconus. *J Refract Surg*. 2009; 25(9): S824–S828. 19772259 10.3928/1081597X-20090813-12

[7] Bottós KM, Schor P, Dreyfuss JL, Nader HB, Chamon W. Effect of corneal epithelium on ultraviolet-A and riboflavin absorption. *Arq Bras Oftalmol*. 2011; 74(5): 348–351. 22183995 10.1590/s0004-27492011000500008

[8] Baiocchi S, Mazzotta C, Cerretani D, Caporossi T, Caporossi A. Corneal crosslinking: riboflavin concentration in corneal stroma exposed with and without epithelium. *J Cataract Refract Surg*. 2009; 35(5): 893–899. 19393890 10.1016/j.jcrs.2009.01.009

[9] Soeters N, Wisse RP, Godefrooij DA, Imhof SM, Tahzib NG. Transepithelial versus epithelium-off corneal cross-linking for the treatment of progressive keratoconus: a randomized controlled trial. *Am J Ophthalmol*. 2015; 159(5): 821–828.e3. 25703475 10.1016/j.ajo.2015.02.005

[10] Al Fayez MF, Alfayez S, Alfayez Y. Transepithelial versus epithelium-off corneal collagen cross-linking for progressive keratoconus: a prospective randomized controlled trial. *Cornea*. 2015; 34: S53–S56. 26266436 10.1097/ICO.0000000000000547

[11] Shalchi Z, Wang X, Nanavaty M. Safety and efficacy of epithelium removal and transepithelial corneal collagen crosslinking for keratoconus. *Eye*. 2015; 29(1): 15–29. 25277300 10.1038/eye.2014.230 PMC4289825

[12] Marcovich AL, Brekelmans J, Brandis A, et al. Decreased riboflavin impregnation time does not increase the risk for endothelial phototoxicity during corneal cross-linking. *Transl Vis Sci Technol*. 2020; 9(6): 4. 10.1167/tvst.9.6.4 PMC7409014 32821501

[13] Gore DM, O'Brart D, French P, Dunsby C, Allan BD. Transepithelial riboflavin absorption in an ex vivo rabbit corneal model. *Invest Ophthalmol Vis Sci*. 2015; 56(8): 5006–5011. 26230765 10.1167/iovs.15-16903

[14] Mastropasqua L, Nubile M, Calienno R, et al. Corneal cross-linking: intrastromal riboflavin concentration in iontophoresis-assisted imbibition versus traditional and transepithelial techniques. *Am J Ophthalmol*. 2014; 157(3): 623–630.e1. 24321474 10.1016/j.ajo.2013.11.018

[15] Hayes S, Morgan SR, O'Brart DP, O'Brart N, Meek KM. A study of stromal riboflavin absorption in ex vivo porcine corneas using new and existing delivery protocols for corneal cross-linking. *Acta Ophthalmol*. 2016; 94(2): e109–e117. 26421680 10.1111/aos.12884 PMC4973833

[16] Rubinfeld RS, Stulting RD, Gum GG, Talamo JH. Quantitative analysis of corneal stromal riboflavin concentration without epithelial removal. *J Cataract Refract Surg*. 2018; 44(2): 237–242. 29526339 10.1016/j.jcrs.2018.01.010

[17] Epstein RJ, Belin MW, Gravemann D, Littner R, Rubinfeld RS. EpiSmart crosslinking for keratoconus: a phase 2 study. *Cornea*. 2023; 42(7): 858–866. 36173242 10.1097/ICO.0000000000003136 PMC10234322

[18] Mazzotta C, Balamoun AA, Chabib A, et al. Transepithelial enhanced fluence pulsed light M accelerated crosslinking for early progressive keratoconus with chemically enhanced riboflavin solutions and air room oxygen. *J Clin Med*. 2022; 11(17): 5039. 36078972 10.3390/jcm11175039 PMC9457355

[19] Watson EB, Dohmen R. Non-traditional and emerging methods for characterizing diffusion in minerals and mineral aggregates. *Rev Mineral Geochem*. 2010; 72(1): 61–105.

[20] Franke MA, Landes T, Seiler TG, et al. Corneal riboflavin gradients and UV-absorption characteristics after topical application of riboflavin in concentrations ranging from 0.1 to 0.5%. *Exp Eye Res*. 2021; 213: 108842. 34793829 10.1016/j.exer.2021.108842

[21] McCarey B, Edelhauser H. In vivo corneal epithelial permeability following treatment with prostaglandin analogs with or without benzalkonium chloride. *J Ocul Pharmacol Ther*. 2007; 23(5): 445–451. 17941807 10.1089/jop.2007.0024

[22] Kissner A, Spoerl E, Jung R, Spekl K, Pillunat LE, Raiskup F. Pharmacological modification of the epithelial permeability by benzalkonium chloride in UVA/riboflavin corneal collagen cross-linking. *Curr Eye Res*. 2010; 35(8): 715–721. 20673048 10.3109/02713683.2010.481068

[23] Raiskup F, Pinelli R, Spoerl E. Riboflavin osmolar modification for transepithelial corneal cross-linking. *Curr Eye Res*. 2012; 37(3): 234–238. 22335811 10.3109/02713683.2011.637656

[24] Novruzlu S, Türkcü ÜÖ, Kvrak I, et al. Can riboflavin penetrate stroma without disrupting integrity of corneal epithelium in rabbits? Iontophoresis and ultraperformance liquid chromatography with electrospray ionization tandem mass spectrometry. *Cornea*. 2015; 34(8): 932–936. 26075452 10.1097/ICO.0000000000000438

[25] Hill J, Liu C, Deardorff P, et al. Optimization of oxygen dynamics, UV-A delivery, and drug formulation for accelerated epi-on corneal crosslinking. *Curr Eye Res*. 2020; 45(4): 450–458. 31532699 10.1080/02713683.2019.1669663

[26] Thaware OC, Huang D. Enrichment of oxygen concentration over simulated corneal surface through noncontact oxygen delivery device. *J Refract Surg*. 2020; 36(9): 613–616. 32901829 10.3928/1081597X-20200611-01 PMC8860776

