# Supplementary figures and images for: Quantitative Measurements of Stromal and Epithelial Riboflavin With a Novel Transepithelial High-Concentration Riboflavin Soak-and-Rinse Protocol
Source: Transl Vis Sci Technol. 2025 Nov 12;14(11):10. doi: 10.1167/tvst.14.11.10 (PMC12614264; doi:10.1167/tvst.14.11.10)

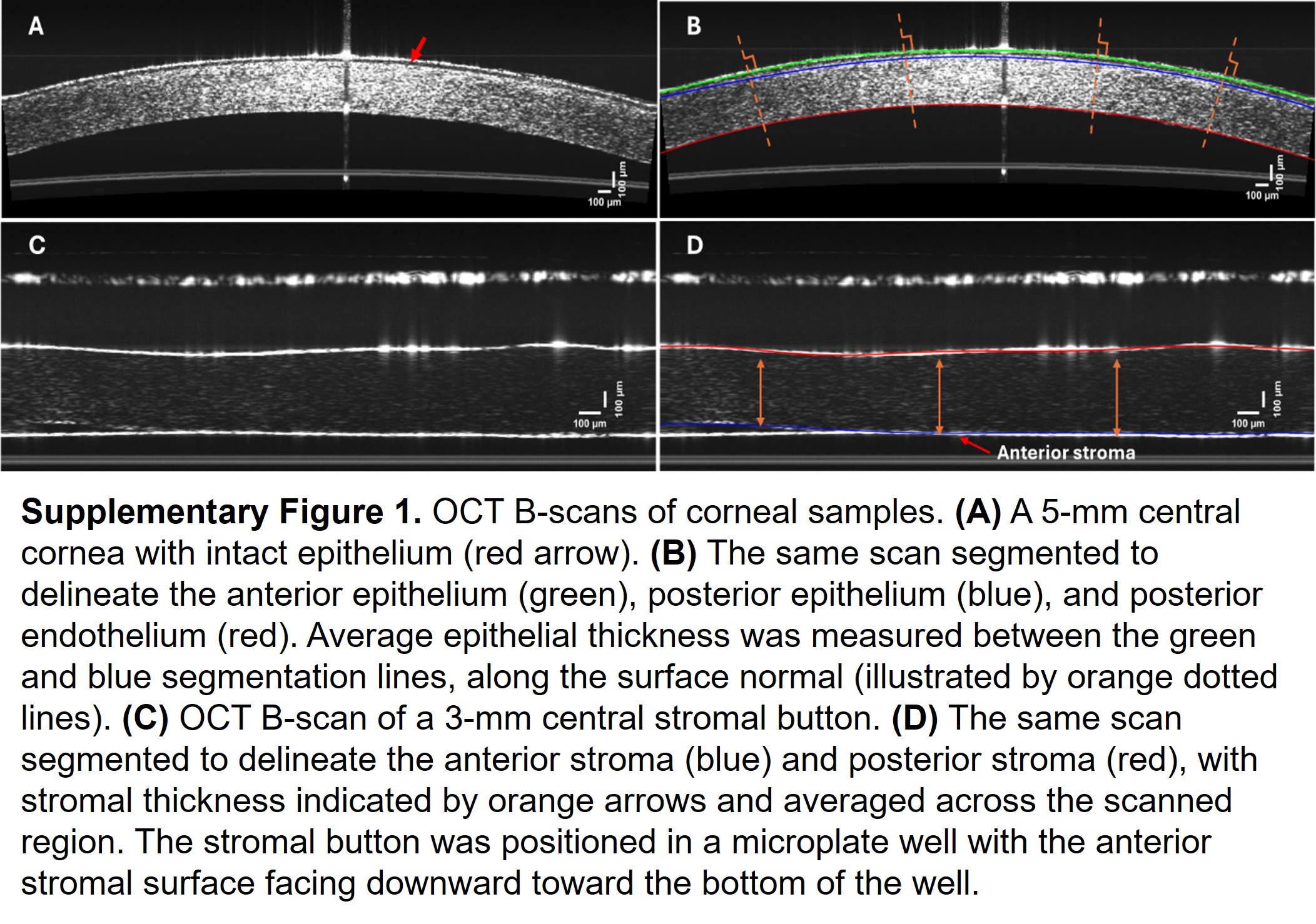

Supplement: Supplement 1 [file tvst-14-11-10_s001.jpg]

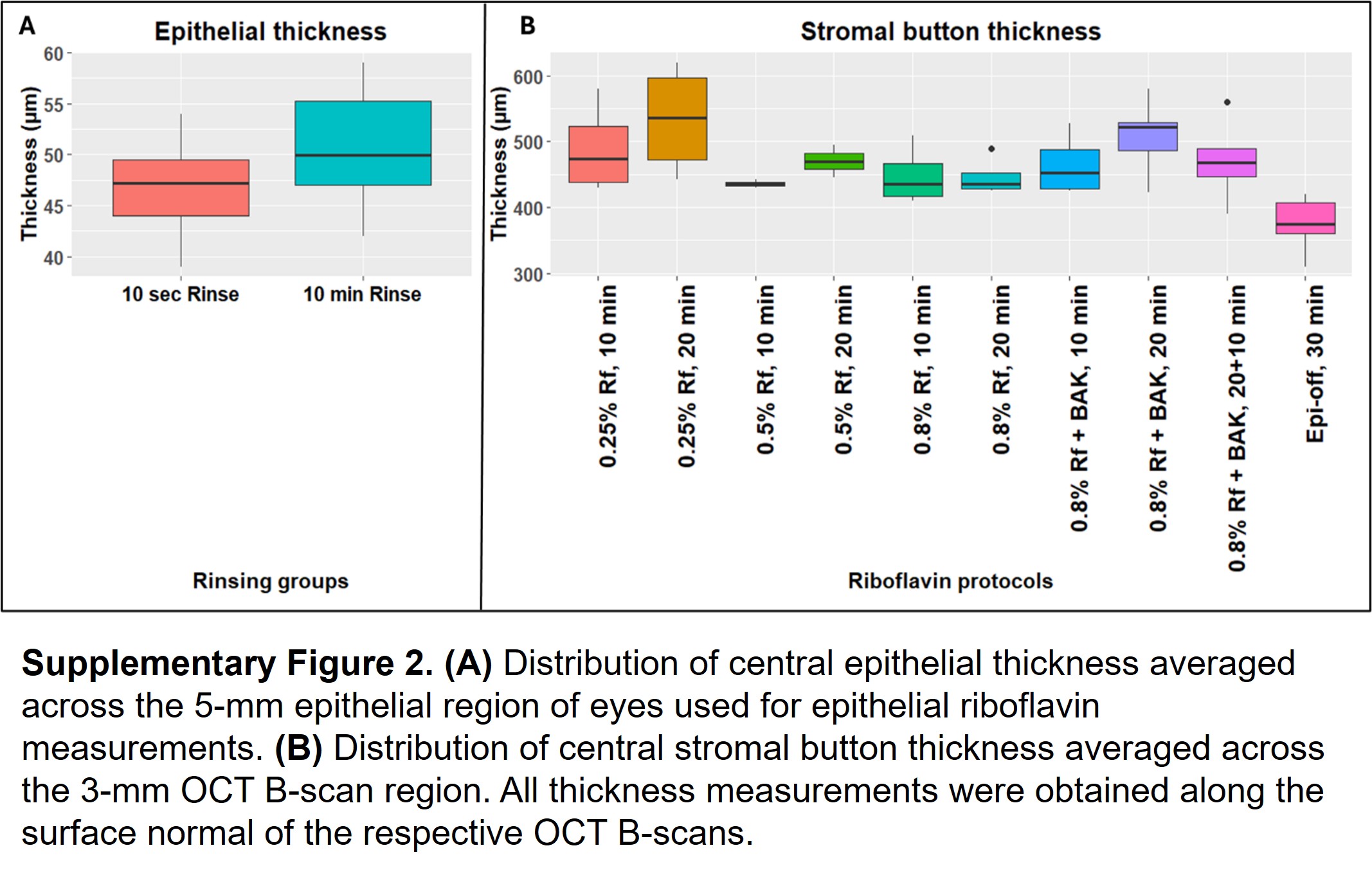

Supplement: Supplement 2 [file tvst-14-11-10_s002.jpg]
